# The Effect of Omega-3 on Mitigating Exercise-Induced Muscle Damage

**DOI:** 10.7759/cureus.81559

**Published:** 2025-04-01

**Authors:** Atiporn Therdyothin, Nacharin Phiphopthatsanee

**Affiliations:** 1 Orthopedics, Police General Hospital, Bangkok, THA; 2 Psychiatry, Faculty of Medicine, King Mongkut's Institute of Technology Ladkrabang, Bangkok, THA

**Keywords:** anti-inflammation, fish oil, muscle function, muscle injury, muscle pain, omega-3, polyunsaturated fatty acids, supplementation

## Abstract

Exercise-induced muscle damage (EIMD) refers to muscle injuries following exercises involving repetitive eccentric muscle contractions. The resultant inflammation and muscle protein leakage into the circulation lead to muscle pain and strength deficit, compromising athletic performance. This narrative review summarizes the current evidence on the effect and mechanism of omega-3 polyunsaturated fatty acids (n-3 PUFA) in potentially mitigating EIMD. Several studies suggested n-3 PUFA’s role in alleviating delayed-onset muscle soreness, particularly in untrained individuals and those receiving higher doses of continuous supplementation. However, its impact on muscle strength attenuation and the reduction of performance post-exercise remains inconclusive. Also unclear are n-3 PUFA’s effects on the reduction of circulating pro-inflammatory substances and muscle proteins. One of the possible mechanisms is its anti-inflammatory property, which involves its ability to incorporate into cell membranes and displace prostaglandin precursor. n-3 PUFA also decreases cyclooxygenase production and can be converted into specialized pro-resolving mediators (SPMs), further reducing inflammation. Moreover, n-3 PUFA’s incorporation into cell membranes alters cell membrane properties, diminishing protein release during muscle breakdown. n-3 PUFA exhibits analgesic effects through SPM-induced modulation of receptors and ion channels, reducing both peripheral and central sensitization. n-3 PUFA also diminishes mitochondrial free radical production and accelerates nerve conduction, thereby improving voluntary muscle activation.

## Introduction and background

Exercise-induced muscle damage (EIMD) is characterized by an initial mechanical stress response during exercise, particularly unfamiliar and repetitive exercises that involve high-intensity eccentric muscle contraction, resulting in myofibrillar disruption due to the disruption of sarcomeres and subsequent increased influx of calcium into muscle cells [1,2]. Breakage of muscle cell membranes is often accompanied by the release of muscle enzymes and proteins, such as creatine kinase (CK) and myoglobin (Mb), which serve as biomarkers for the extent of muscle damage [3]. This initial injury triggers a secondary inflammatory response. Through the activation of transcription factors like nuclear factor kappa B (NF-κB) and mitogen-activated protein kinase (MAPK), cascades of proinflammatory cytokines, i.e., prostaglandins (PGs), leukotrienes, IL-6, tumor necrosis factor (TNF), and IL-1β, are released into local tissues and circulation [4]. The secretion of cytokines and activation of transcription factors then contribute to a cascade of cellular events, including the production of reactive oxygen species (ROS) from injured muscle cells as well as neutrophils and macrophages recruited to the damaged area [1,4]. EIMD manifests in various symptoms, including delayed-onset muscle soreness (DOMS), reduced muscle strength and power, and diminished overall muscle performance, all of which impact exercise performance. DOMS typically emerges within 24 hours post-exercise, peaks around 24-48 hours, and subsides within five to seven days [5,6].

Currently, common remedies to alleviate DOMS include physical therapy such as massage [7], cryotherapy [8,9], and pharmacologic agents including nonsteroidal anti-inflammatory drugs (NSAIDs) [10,11]. With increasing recognition of the potential involvement of both inflammatory responses and the generation of ROS and free radicals during and after exercise in DOMS, nutrition-based interventions targeting post-exercise inflammation and oxidative stress responses have gained significant attention [3,12], with a focus on omega-3 polyunsaturated fatty acids (n-3 PUFA) [6].

n-3 PUFA is one of the two groups of essential fatty acids, with the other being n-6 PUFA. n-3 PUFA is characterized by a double bond on the third carbon atom from the terminal methyl group [13]. This class is primarily divided into three essential components: alpha-linolenic acid (ALA), eicosapentaenoic acid (EPA), and docosahexaenoic acid (DHA) [14]. DHA is the predominant n-3 PUFA in cell membranes and is found in all organs, including the spleen, liver, muscles, and heart, with particularly high concentrations in neural structures like the brain and retina [15]. ALA, albeit less potent, can be converted to EPA and subsequently DHA through enzymatic reactions, specifically desaturation and elongation [13]. The conversion efficiency of ALA to EPA and DHA is relatively low, prompting the recognition of the importance of direct consumption of EPA- and DHA-rich sources. These sources include oily fish [16] such as salmon [17], mackerel, sardines, herrings, and trout, as well as alternative sources like algae [18] and krill [19].

The n-6 PUFA linoleic acid is converted to gamma-linolenic acid, which is further elongated and desaturated to dihomo-gamma-linolenic acid and arachidonic acid (AA) using the same enzymes as the n-3 PUFA pathways [13]. Therefore, the two pathways compete with each other and influence the balance of inflammatory and anti-inflammatory mediators produced from these fatty acids. The metabolism of AA leads to the production of pro-inflammatory mediators such as PGE2, thromboxane A2, and leukotriene B4. Diets consisting of a high amount of n-6 PUFAs can potentially inhibit the conversion of ALA to anti-inflammatory EPA and DHA and have been linked to chronic inflammatory conditions [20]. Given n-3 PUFA’s recognized anti-inflammatory properties and established benefits for cardiovascular health without significant side effects [21,22], EPA and DHA are also frequently taken in the form of nutritional supplements.

Beyond general health benefits, n-3 PUFAs have gained attention in the context of athletic performance and recovery. The International Olympic Committee’s consensus statement on dietary supplements suggests the potential benefits of fish oil consumption [23]. The International Society of Sports Nutrition also published a position statement supporting the use of n-3 PUFA in athletes, as n-3 PUFA has been shown to improve endurance and cardiovascular function during aerobic exercise and may also enhance recovery from EIMD [24].

Despite growing interest in the role of n-3 PUFAs in exercise recovery, existing research on the impact of fish oil on recovery from EIMD yields inconsistent findings [6,25-27]. Current research is limited by variability in study design, differences in supplementation protocols, and the lack of standardized outcome measures. In this narrative review, we will explore the benefits of n-3 PUFA supplementation on different aspects of EIMD, which are DOMS, muscle strength, and performance, as well as the release of muscle damage biomarkers and inflammatory cytokines. We will also discuss the mechanism by which n-3 PUFA affects recovery from exercise.

## Review

The effect of n-3 PUFA on DOMS

In a systematic review [28], the supplementation of n-3 PUFA demonstrated an ability to alleviate DOMS following EIMD with moderate heterogeneity. The observed effects remained consistent across varying doses (<1,000 g/day and >1,000 g/day) and durations of supplementation (less than seven days and more than seven days). Nevertheless, the authors stated that the improvement in DOMS did not surpass the minimal clinically important difference for pain reduction, defined as 1.4 out of 10 on the Visual Analog Scale (VAS) [28,29]. In other words, this implies that the reduction in pain scores may not reach clinical significance. Noteworthily, the pain assessments in this meta-analysis were conducted at 48 hours post-exercise, potentially providing a limited snapshot of the overall impact. A more comprehensive evaluation should include assessments at different time points to capture the overall effect of n-3 PUFA on DOMS, i.e., at 24-36 hours, which are also the peak of DOMS [30], or at 72 or 96 hours post-exercise to evaluate the effect of supplementation on recovery of EIMD [11,31]. Moreover, the time required for pain to subside may offer a further understanding of n-3 PUFA's benefits in pain recovery. It is important to be aware of the heterogeneity of the outcomes in the included studies, considering the diverse nature of exercises in various trials, including differences in type, intensity, and targeted muscle groups. Performing subgroup analyses based on the anatomical region of exercise is crucial, as different muscle groups may vary in terms of response to both exercise [32] and nutritional supplementation [33].

Tsuchiya et al. [34] reported that eight-week n-3 PUFA supplementation significantly reduced VAS scores on day 5 post-exercise compared to a placebo, bringing pain scores down to almost the pre-exercise level, while the placebo group showed little change. However, trials with shorter supplementation durations did not yield significant differences in DOMS [35-37]. VanDusseldorp et al. [35] investigated the impact of varying fish oil doses in resistance-trained individuals after 80 eccentric squats. Only the group receiving 6 g per day of supplementation exhibited lower DOMS at 2, 48 hours, and 72 hours post-exercise after seven weeks of supplementation. The groups receiving lower doses (2 g and 4 g) of supplementation did not differ in the perceived muscle soreness compared to the placebo group. Notably, the effective dosage in this study was higher than in other trials [6,26,34], suggesting a potential discrepancy in the effectiveness based on individuals’ training status, i.e., trained individuals might benefit less from n-3 PUFA supplementation compared to untrained counterparts [38]. In a randomized controlled crossover trial involving amateur endurance athletes, a 10-week supplementation of 2.1 g/d DHA and 240 mg/d EPA resulted in lower VAS scores at all time points compared to the placebo group [39]. The reduction in pain paralleled the reduction in serum lactate dehydrogenase-5 (LDH-5), which signifies muscle damage [40]. However, the robustness of the findings is limited by the high dropout rate of 42%. In a trial involving 14 healthy untrained males, 3 g/day of n-3 PUFA supplementation for four weeks significantly decreased VAS at 24 hours post-exercise after 60 minutes of downhill running, but not at later time points [6]. A randomized controlled trial found that seven-week supplementation with 4 g of EPA or 4 g of DHA significantly reduced muscle soreness at 48 hours, while the combined 4 g EPA+DHA group showed no significant effect. This suggests that combining EPA and DHA may diminish their individual benefits for EIMD recovery [41].

A shorter duration of supplementation produced less promising results. In a randomized controlled trial, 27 physically active males were assigned to three groups: placebo, low-EPA fish oil (low EPA), or high-EPA fish oil (high EPA) [27]. In the high EPA group, participants consumed fish oil capsules containing 750 mg EPA and 50 mg DHA, with the number of capsules proportional to their body mass (1 g/10 kg body mass). The low EPA group took the same number of capsules, but each contained 150 mg EPA and 100 mg DHA. Despite these variations in EPA and DHA contents, perceived muscle soreness was similar across groups. Another randomized controlled trial in healthy young women revealed that three days of high-dose n-3 PUFA (3,200 g) supplementation did not improve DOMS after 100 eccentric knee contractions [26]. Similarly, protein, omega-3 fatty acid, and creatine supplementation for five days did not alter perceived muscle soreness in resistance-trained females [42]. The explanation provided was that longer-term supplementation is required for n-3 PUFA to incorporate into muscle cell membranes [43,44], altering their permeability and reducing susceptibility to inflammation [45]. Additionally, hormonal fluctuations during the menstrual cycle were also considered to potentially influence DOMS, as DOMS was generally higher in the mid-luteal and early follicular phases [46].

Considering these diverse findings, the overall impact of n-3 PUFA supplementation on DOMS following EIMD tends to be positive, but this remains controversial. Further investigations are required to allow translation of statistical significance into clinically meaningful improvements. Overall, more substantial benefits may be seen in untrained individuals receiving longer durations of pre-exercise supplementation, and the effectiveness may depend on dosage, stimulus intensity, or training status.

Muscle strength and function

The assessment of muscle strength and function presents inherent challenges due to its diverse and complex nature. According to a recently published systematic review, n-3 PUFA supplementation after EIMD did not result in any changes in the maximal isokinetic torque; however, a notable enhancement in the maximal isometric torque was observed with the administration of both pure n-3 PUFA and n-3 PUFA combined with other supplements [47]. A larger effect was observed in the longer duration of supplementation. Conversely, a meta-analysis did not reveal a significant improvement in isometric muscle strength following EIMD with n-3 PUFA supplementation [28].

In a randomized controlled trial conducted by Tsuchiya et al. [34], a supplementation regimen of 600 mg EPA and 260 mg DHA/day for eight weeks and five days attenuated the maximal voluntary contraction (MVC) loss from immediately post-exercise to five days after EIMD in 16 healthy men. The EIMD was induced by six sets of 10 eccentric contractions of biceps brachii at 100% MVC. The range of motion (ROM) of the elbow was significantly better in the n-3 PUFA group, returning to pre-exercise level after two days, while the ROM of the placebo group remained diminished. n-3 PUFA also prevented swelling and muscle stiffness of the arm after eccentric contraction. However, the study was limited by a very small number of participants.

Another study by Tsuchiya et al. [48] emphasized the importance of supplementation duration by investigating the effects of four weeks of identical n-3 PUFA supplementation following EIMD. They did not find a significant improvement in muscle strength and power. However, an immediate post-exercise ROM was improved, which could be explained by a decreased inflammatory response in myofibrils, as well as an attenuated elevation of cytoplasmic calcium level and muscle swelling [48-50].

In another clinical trial, 2, 4, and 6 g of n-3 PUFA supplementation for seven weeks did not outperform placebo in restoring the MVC of knee extensors and 40-yard sprint time [35]. Only 6 g of supplementation could restore vertical jump height to pre-injury status from one hour post-exercise, while lower doses of n-3 PUFA supplementation did not outperform placebo, suggesting the effect of a higher dose of fish oil in improving muscle performance. Similarly, a randomized crossover controlled trial in 15 male athletes using 2.34 g of n-3 PUFA supplementation a day found no improvement in knee isokinetic strength, peak torque flexion, and peak torque extension after an ECC protocol containing squats and vertical jumps [39]. However, other measures such as VAS and serum LDH were markedly improved in the n-3 PUFA supplementation group. The author suggested that the insignificant improvement in strength deficit was due to the lack of specified measurements of torque and strength. Each exercise uses a different muscle group; therefore, the measurement of post-exercise torque or strength deficit should be performed on the same muscle group in a similar position.

A study involving 60 minutes of downhill running found no significant differences in immediate leg extension MVIC and peak power between the n-3 PUFA and placebo groups. Both groups returned to baseline performance at 24 hours after exercise [6]. The quick recovery time in both groups was suggestive of an inadequate induction of EIMD, potentially compromising the effect of n-3 supplementation.

On the contrary, a randomized controlled trial conducted by Loss et al. [26] in young women receiving three days of high-dose n-3 PUFA supplementation demonstrated no improvement in knee extensor isokinetic and isometric peak torques. Similarly, short-term consumption of fish oil did not improve the isokinetic muscle function of the knee extensor after 100 plyometric drop jumps compared to a placebo in physically active men [27]. A similar finding was observed for countermovement jump performance. Surprisingly, a post-EIMD consumption of high-EPA fish oil alleviated the reduction in squat jump performance compared to low-dose EPA and placebo. Moreover, participants receiving high EPA fish oil returned to their baseline performance level within 24 hours, while the rest still experienced a performance deficit of 12%, suggesting the effect of high EPA in facilitating performance recovery. Because jump performance requires complex activation of muscle groups, the authors suggested that EPA supplementation allowed for greater voluntary activation of the muscles due to the improvement of peripheral neuromuscular function in experimental athletic situations [51].

The timing of supplementation might also impact the effect on muscle performance. A randomized controlled trial in 15 female futsal players found that taking 1 g of fish oil and 30 g of whey protein two hours before EIMD significantly improved muscle strength and power compared to placebo and post-EIMD supplementation [52].

Overall, the effect of N-3 supplementation on muscle strength and performance remains inconclusive, and most findings from the studies were insignificant. The heterogeneity of results was attributable to the difference in the measurement of muscle strength and performance, as well as the difference in nature from each muscle group.

The effect of n-3 PUFA supplementation on blood markers of EIMD

Eccentric contractions induce tension within the muscle, leading to the disruption of muscle structures and contributing to muscle injuries [53]. The local response to muscle injury encompasses the release of biomarkers indicative of muscle damage, such as Mb or CK, as well as the production of inflammatory markers, including CRP, TNF-α, IL-1, and IL-6, released from the injured muscle cells and surrounding leukocytes [54].

Markers for Muscle Damage

A recent meta-analysis evaluating the effect of n-3 PUFA supplementation on blood markers of EIMD found that n-3 PUFA was associated with significantly lower CK concentration, albeit with high heterogeneity [55]. A similar effect was observed across the trials, regardless of the duration of supplementation, except for the trial using an acute dose of n-3 PUFA after exercise. This can be partly explained by the time needed for n-3 PUFA to incorporate into cell membranes, preventing leakage of muscle proteins [56,57], and demonstrating anti-inflammatory effects [45]. This meta-analysis also revealed a significant decrease in CK in only untrained participants as opposed to trained participants [55], suggesting greater efficiency in untrained individuals. This result was also replicated in another recent trial involving trained young males [58]. The differential effect may be explained by the varying degree of injury incurred by EIMD [59]. However, subgroup analysis based on the doses was not performed. In contrast, another two systematic reviews had reservations about the effect of n-3 PUFA on the inhibition of CK release, concluding that no consensus could be reached [28,60]. The meta-analysis also identified a significant change in Mb concentrations from a total of 86 participants [55]. Subgroup analysis showed that n-3 PUFA supplementation significantly lowered Mb concentrations in the RCTs with 48 and 72-hour measurements of Mb after exercise. It is important to note that all trials measuring Mb levels involved a supplementation duration of over one month and were all conducted with untrained participants. A subgroup analysis of the trials with longer than one month of supplementation also revealed a significant reduction in LDH concentration at 24 and 48 hours after EIMD.

A recent clinical trial conducted in healthy young men revealed that four weeks of EPA-rich fish oil supplementation significantly reduced CK level when compared to placebo at day 3 after EIMD, suggesting its role in preventing muscle damage after ECC of the biceps brachii [61]. The reduction in CK was parallel to the improvement in the ROM of the associated joint, which was also explained by decreased inflammation and muscle damage [49]. On the contrary, the reduction in CK was not observed in another trial with an identical protocol, despite a longer supplementation period and a significant improvement in muscle soreness and ROM [48]. A randomized crossover-controlled trial in 15 male athletes found a significant reduction in serum LDH-5 after 10 weeks of n-3 PUFA supplementation compared to placebo. The study also found a reduction in IL1b and CPK at 24 hours after 48 half squats and 80 deep jumps. The exercise used in this study was more intensive than many other studies that used elbow flexions and therefore might reveal clearer effects of n-3 PUFA in alleviating muscle damage [39].

Markers for Inflammation

The extent to which n-3 PUFA supplementation can reduce CRP after EIMD remains questionable. A study in overweight and obese males found no change in serum CRP levels in the group receiving 4 g of fish oil supplementation for four weeks compared to the control group after high-intensity interval training; however, the study found a significant increase in white blood cell count in the control group immediately after high-intensity interval training, while the count remained unchanged in the supplementation group [62]. A recent systematic review [63] identified three studies measuring CRP levels, two of which demonstrated no change in CRP levels [36,63]. Of note, in one study, CRP was measured in saliva as opposed to serum [63]. Moreover, no change in CRP level was detected after exercise in either group, questioning the adequacy of exercise intensity and sensitivity of salivary CRP. The same systematic review and meta-analysis found 7 studies comparing IL-6 levels after EIMD, 4 of which reported a significant decrease in IL-6 in n-3 PUFA groups [28]. Lastly, a reduction in TNF-α was observed in only one out of three studies with n-3 PUFA supplementation after EIMD [28].

However, another trial conducted in 14 healthy young men found no significant effect of n-3 PUFA on IL-6, CK, and TNF-alpha levels after 60 minutes of downhill running. In this trial, IL-6 returned to the baseline levels at 24 hours, while CK was increased in all groups. TNF-alpha did not significantly differ from baseline in both groups. However, questions were raised about the rigorousness of the exercise protocol [6].

In summary, the effect of n-3 PUFA on blood markers is still inconclusive, with heterogeneity stemming from the variation in the doses of EPA and DHA, the duration of supplementation, exercise protocol, the muscle groups involved, and the training status of the participants. The benefits in the reduction of muscle damage markers tend to be more pronounced in untrained participants receiving a longer duration of supplementation.

**Figure 1:**
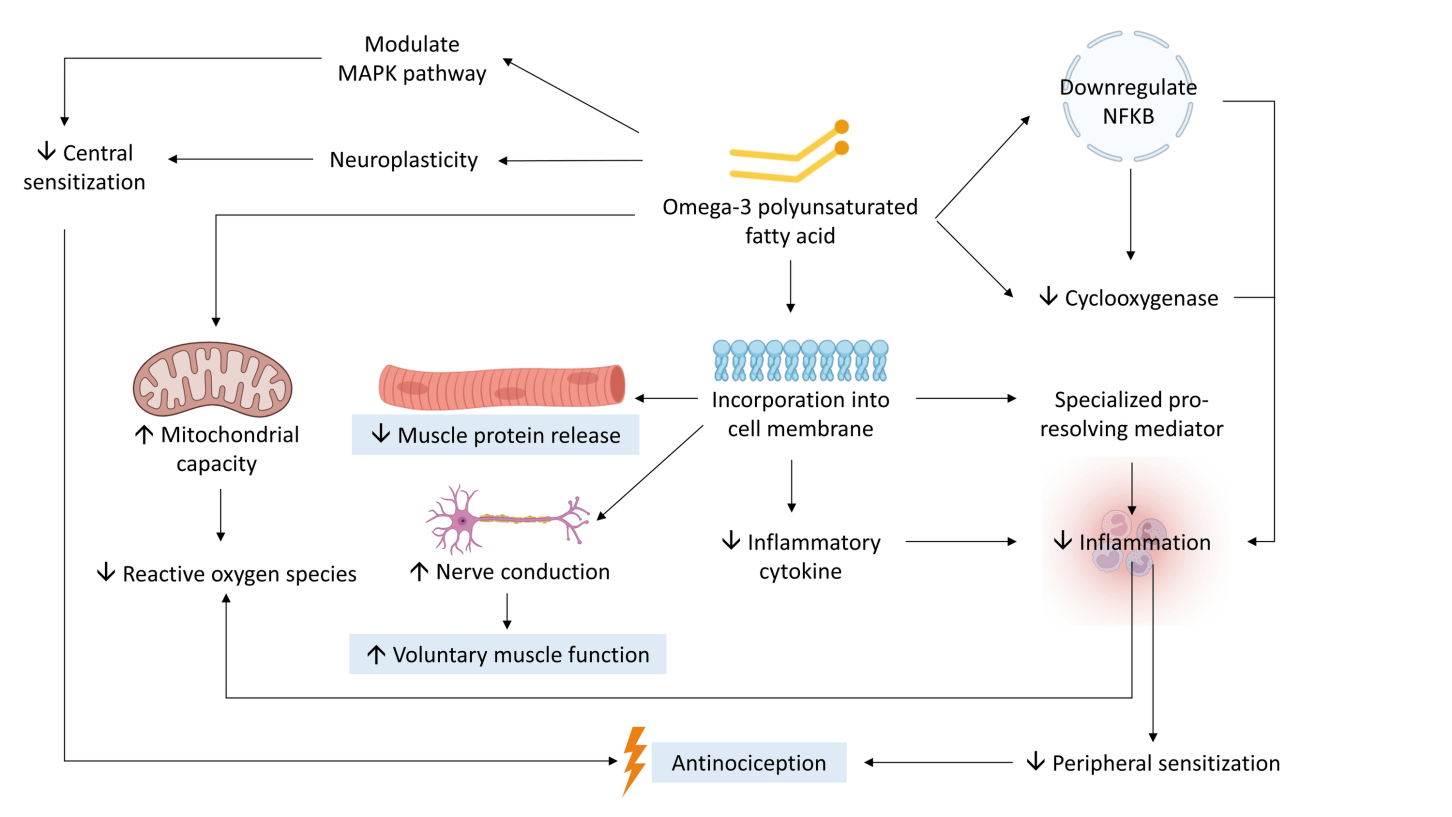
Schematic representation of the mechanism of n-3 PUFA on alleviation of EIMD n-3 PUFA can be incorporated into cell membranes and displace precursors for potent inflammatory cytokines, leading to its anti-inflammatory effect. It can also be metabolized into SPMs, which further reduce inflammation. n-3 PUFA can directly reduce the production of COX, a key enzyme for inflammation, and indirectly by downregulating NF-κB. Its incorporation into muscle cell membrane also alters membrane property and permeability and subsequently prevents leakage of muscle proteins into circulation during EIMD. Moreover, altered membrane properties affect nerve conduction, resulting in greater voluntary muscle activation. n-3 PUFA enhances mitochondrial capacity and therefore reduces the emission of ROS. N-3 PUFA also has an anti-nociception effect, which relies on the reduction of both central and peripheral sensitization. Central sensitization can be tempered through n-3 PUFA’s modulation of MAPK and increased neuroplasticity. Lastly, diminished peripheral sensitization is attributed to decreased inflammation. COX, cyclooxygenase; EIMD, exercise-induced muscle damage; MAPK, mitogen-activated protein kinase; n-3 PUFA, omega-3 polyunsaturated fatty acid; NF-κB, nuclear factor kappa B; ROS, reactive oxygen species; SPM, specialized pro-resolving mediator

Mechanism of n-3 PUFA on attenuating EIMD

n-3 PUFA may attenuate EIMD through various interrelated mechanisms (as shown in Figure 1). n-3 PUFAs’ ability to incorporate into cell membranes enables them to exert anti-inflammatory properties and simultaneously alter membrane permeability, resulting in reduced leakage of muscle enzymes [45]. n-3 PUFAs also exhibit the capacity to modulate pain perception and sensitization [64]. The anti-oxidative properties of n-3 PUFAs are manifested through the scavenging of free radicals and the induction of enzymatic reactions involved in the destruction of these reactive species [43,65]. Lastly, n-3 PUFAs have been shown to enhance neural activation and nerve conduction [66], leading to improved voluntary muscle contraction.

Anti-Inflammatory Property

The most important mechanism by which n-3 PUFA mitigates EIMD is probably through anti-inflammation [45]. Its anti-inflammatory potency initiates from the incorporation of EPA and DHA into cell membranes, modifying the property of lipid rafts on cell membranes and leading to an alteration of both membrane composition and intracellular signaling [67]. Unlike NSAIDs, which directly block cyclooxygenase (COX) enzyme activity and hence inhibit the production of inflammatory eicosanoids, n-3 PUFA competes with AA in the COX pathway, reducing but not completely blocking the production of pro-inflammatory mediators [68]. EPA and DHA displace membrane-bound AA, the main substrate for the production of potent pro-inflammatory mediators, particularly PGs by COX enzyme [69]. Instead, EPA and DHA are metabolized into eicosanoids with lower inflammatory potency such as PGE3, mitigating inflammation [70]. Studies have confirmed the dose-response effect of n-3 PUFA supplementation in reducing PGE2 in humans [71]. Due to the mechanism differences, NSAIDs provide faster pain relief by directly blocking COX enzymes, while n-3 PUFAs require more time, sometimes weeks or months, to yield effects. Consequently, n-3 PUFA supplementation is considered a preventative approach rather than an immediate alternative to NSAIDs for acute inflammation [72].

Moreover, n-3 PUFA downregulates NF-κB [72], one of the most important transcription factors associated with inflammation, resulting in lower COX enzyme production [73]. n-3 PUFA also activates peroxisome proliferator-activated receptor gamma (PPAR-γ) [73,74], which helps to reduce inflammatory cytokine production and further interfere with the translocation of NF-κB into the nucleus [75]. EPA and DHA also enhance G protein-coupled receptor GPR120 and its subsequent inhibition of NF-κB [76], thereby inhibiting COX production.

Apart from their intrinsic characteristics, EPA and DHA can also be metabolized into pro-resolving and anti-inflammatory lipid mediators (specialized pro-resolving mediators (SPMs)), such as resolvins, protectins, and maresins [77]. Resolvin E1 (RvE1), resolvin D1 (RvD1), and protectin D1 inhibit transendothelial migration of neutrophils, preventing leukocyte aggregation at the site of inflammation [78]. Protectin D1 and RvD1 decrease TNF‐α and IL‐1β production [78]. RvD1 can also reduce inflammation by inhibiting the activation of the MAPK pathway, decreasing NF-kB expression, and increasing the production of NF-kB inhibitor protein [79,80]. RvD2 inhibits the nuclear translocation of NF-kB, hence reducing its downstream COX production and inflammation [81]. Moreover, SPM has also been demonstrated to reduce pain sensitization [64], which will be discussed later.

Altering Membrane Permeability of Muscle Enzymes

EPA and DHA can be incorporated into muscle cell membranes and enhance their fluidity, flexibility, and stability, therefore lowering muscle enzyme release. Incongruence in serum CK concentration and inflammatory cytokines after EIMD in participants receiving n-3 PUFA supplementation indicated the effect of n-3 PUFA in altering membrane permeability [55,82] and lowering the release of muscle damage markers into the circulation without the effect on sarcomere disruption [83,84]. This suggests the benefits of omega-3 consumption in improving muscle cells’ membrane stability and lowering the leakage of muscle enzymes. Apart from the muscle cell membrane, n-3 PUFA is also incorporated into the sarcolemmal and mitochondrial membranes, potentially affecting membrane protein localization and modifications and influencing sarcolemmal substrate transport, mitochondrial function, and protein synthesis [85].

Anti-Oxidative Property

Another mechanism by which n-3 PUFA supplementation might alleviate EIMD is through its anti-oxidative properties. A trial involving 20 males supplemented with six weeks of either fish oil or placebo found lower levels of plasma thiobarbituric acid reactive substances and hydrogen peroxide-stimulated lymphocyte DNA damage after performing 200 eccentric knee contractions in participants receiving fish oil [37]. However, no changes in CK, endogenous lymphocyte DNA damage, muscle soreness, and MVC were observed. The study suggested that fish oil supplementation may reduce exercise-induced oxidative stress and decrease the vulnerability to oxidative damage. EPA and DHA may function as free radical scavengers, decreasing thiobarbituric acid reactive substances and ROS-induced DNA damage [86]. EPA was found to induce catalase activity in skeletal muscle cells and increase the expression of manganese superoxide dismutase and catalase enzymes [87].

Apart from its anti-inflammatory properties, n-3 PUFAs’ ability to be incorporated into cell membranes and displace AA is also associated with lower lipid oxidation through a reduction of PGE-2 production [88], a process by which free radicals are generated [89]. Hence, n-3 PUFA’s ability to reduce inflammation can also lead to decreased oxidative stress.

n-3 PUFA has been found to reduce ROS generation through enhanced mitochondrial function in older adults [90]. EPA’s anti-inflammatory property was found to be related to an increased expression of transcriptional regulators of mitochondrial biogenesis. Older adults receiving four months of 3.9 g/day of n-3 PUFA supplementation exhibited a 20-25% reduction in mitochondrial hydrogen peroxide production in muscle cells without altering mitochondrial respiration rates, suggesting that n-3 PUFA can reduce intrinsic mitochondrial ROS generation [65]. This antioxidative potential contributes to the protection of cellular structures and functions from oxidative stress induced by exercise. Herbst et al. [43] found that n-3 PUFA supplementation increased the EPA and DHA contents in mitochondrial membranes and enhanced mitochondrial capacity by elevating maximal respiration and adenosine diphosphate (ADP) sensitivity. The author suggested that an enhanced ADP sensitivity may improve the efficiency of adenosine triphosphate resynthesis during exercise, contributing to omega-3-induced improvements in skeletal muscle contraction efficiency in animals [91] and humans [92].

Anti-Nociception and Pain Modulation

n-3 PUFA can attenuate EIMD, particularly DOMS, by modulating pain pathways and perceptions. The attenuation of both localized and systemic inflammatory processes attributable to n-3 PUFA results in diminished peripheral and central pain [93]. n-3 PUFAs reduce the production of inflammatory cytokines and eicosanoids [69], both of which directly activate peripheral nociceptors to evoke spontaneous pain [94] and heighten nociceptor sensitivity through modulation of various ion channels, such as voltage-gated sodium channels [95] and transient receptor potential (TRP) channels [96]. In other words, n-3 PUFAs can help to reduce localized inflammation and peripheral pain. Proresolving mediators, such as RvD1, help to alleviate inflammation and pain by decreasing TNFα and IL-1β levels in arthritic joints [97,98]. RvD2, RvE1, RvD1, and MaR1 modulate peripheral sensitization and also spinal cord pain transmission by inhibiting TRP vanilloid 1 currents in neurons [96,99,100]. Moreover, RvE1 and NPD1 activate G protein-coupled receptors and play a crucial role in pain resolution [101,102]. RvE1 also binds to chemokine-like receptor 1, resulting in analgesic effects [103].

Central sensitization is induced by the activation of N-methyl-D-aspartate receptors and MAPK [104,105]. The latter is modulated by n-3 PUFA intake, mitigating central sensitization and neuropathic pain [106]. n-3 PUFA can also further reduce central sensitization by increasing neuroplasticity and neurogenesis [107,108].

Enhancement of Nerve Conduction

As an important component of the myelin sheath, axon terminal, and cell membranes, n-3 PUFA may alter cellular membrane composition and fluidity, augmenting nerve conduction, lowering nerve resistance, and improving ion channel function regulation of mitogen-activated kinase transcription factors [109,110]. n-3 PUFA modulates action potential transmission and therefore enhances muscle activation and EMG [100,111-113]. The improvement in neurological responses in mammals and humans has been suggested to be attributable to n-3 PUFAs’ ability to inhibit the delay of nerve conduction velocity (NCV) [114-116].

In an experiment in diabetic rats, eight weeks of fish oil supplementation (EPA and DHA) at a dose of 0.5 g/kg/day resulted in a change in sciatic nerve plasma membrane fatty acid composition [115]. Moreover, sodium-potassium ATPase activity was restored to non-diabetic levels after fish oil supplementation [114]. The changes were parallel to an improvement in NCV in rats with diabetic neuropathy. DHA alone was shown to totally prevent the decrease in NCV and nerve blood flow in diabetic rats compared to the non-supplemented diabetic group [117]. Furthermore, DHA levels in sciatic nerve membranes were proportional to NCV. Similar to exercise, DHA supplementation was shown to increase NCV, brain theta, alpha, and beta activities, compensating for the effects of nerve hypoxia in rats [116].

The findings were consistent in clinical trials. Eight-week supplementation of EPA and DHA in active young men inhibited musculocutaneous NCV latency following a single session of maximal elbow flexor eccentric contractions. Improvements were also observed in other parameters of EIMD, including a decrease in DOMS and an increase in elbow ROM and MVC torque [118]. The ergonomic potential of n-3 PUFA was explored in a study by Lewis et al. [51], in which male athletes were supplemented with 21 days of daily N-3 in the form of 5 ml seal oil containing 375 mg EPA, 230 mg DPA, and 510 mg DHA. n-3 PUFA supplementation resulted in a 20% increase in quadriceps EMG. Participants receiving the supplement reported less fatigue, along with the observed increase in muscle activation, indicating improved peripheral neuromuscular function. This neural modulation suggests that the benefits of n-3 PUFAs extend beyond a mere anti-inflammatory and antioxidative role, encompassing a direct impact on the neuromuscular system.

In summary, the effects of n-3 PUFAs in attenuating EIMD involve anti-inflammatory actions, alteration of membrane permeability, modulation of pain perception, antioxidative capabilities, and enhancement of neural activation. n-3 PUFA’s variety of mechanisms highlights its potential as a multimodal intervention in mitigating the physiological consequences of EIMD.

## Conclusions

EIMD is a consequence of excessive eccentric muscle contractions, which results in inflammation and muscle protein leakage into the systemic circulation. n-3 PUFA has a promising role in reducing DOMS, particularly in untrained individuals, and its efficacy appears to be related to a longer duration of supplementation. Nevertheless, due to methodological heterogeneity in the studies included, its impact on post-exercise muscle strength attenuation and performance reduction remains inconclusive. Similarly, the effect of n-3 PUFA on pro-inflammatory substances and muscle proteins after EIMD requires further exploration. This comprehensive review delineates the anti-inflammatory properties of n-3 PUFA, including its ability to displace the precursor or potent pro-inflammatory cytokines, to reduce COX, and to transform into SPM. n-3 PUFA can also modulate receptors and ion channels, demonstrating analgesic properties. These mechanisms, coupled with the mitigation of mitochondrial free radical production and acceleration of nerve conduction, suggest a role of n-3 PUFA in EIMD attenuation.
